# Knowledge and awareness of tuberculosis among Roma population in Belgrade: a qualitative study

**DOI:** 10.1186/1471-2334-11-284

**Published:** 2011-10-24

**Authors:** Dejana S Vukovic, Ljudmila M Nagorni-Obradovic

**Affiliations:** 1 Institute of Social Medicine (Dr Subotica 15), Faculty of Medicine Belgrade (11000), Serbia; 2 Clinics for Pulmology (Koste Todorovica 26), Clinical Centre of Serbia, Faculty of Medicine Belgrade (11000), Serbia

## Abstract

**Background:**

Tuberculosis (TB) remains an important health problem in the Roma population in Serbia. Recent studies have highlighted the importance of increasing awareness of TB and reducing the associated stigmas to reduce the incidence of TB and enable earlier diagnosis and effective treatment. This study investigated the knowledge and beliefs about transmission, symptoms and treatment of TB as well as attitudes towards patients with TB among the Roma population in Belgrade.

**Methods:**

The focus-group method was considered to be appropriate for investigating knowledge and beliefs about TB. A total of 24 Roma people aged 19-55 years participated in three focus-group discussions.

**Results:**

All participants knew that TB was a pulmonary disease and could be contagious. Saliva was the most commonly mentioned mode of transmission. Some individuals thought, albeit hesitantly, that TB could be transmitted by shaking hands with an infected individual. Of factors contributing to TB, participants mentioned bad living conditions, low quality and lack of food, and stress. Participants quoted chest pain, cough, haemoptysis, loss of appetite, loss of weight, weakness and sweating as basic symptoms of TB. Participants believed that effective treatment should include resting, taking prescribed medicines, inhaling fresh air and eating "strong" food such as bacon and pork; these approaches were considered as important as taking antibiotics). In addition, participants mentioned that they use some folk medicines.

Relatives and friends, and to a lesser extent television, were the main sources of information about TB. Participants most appreciate personal contact with doctors as a source of information.

**Conclusions:**

We concluded that participants were aware of the seriousness TB as well as some of the modes of transmission; however, they had some misconceptions. An important finding was the confidence in doctors expressed by the Roma people.

## Background

Tuberculosis (TB) remains an important health problem in Serbia. There has been no reduction in the incidence from 1994 to 2004; the incidence in 1994 was 34 per 100,000 and in 2004 it was 35 per 100,000 inhabitants. Age-standardized mortality rate in 2004 was 2.45 per 100 000 inhabitants. Researchers also reported an increased mortality rate in the period 1982-1996, probably caused by poor economic conditions and migration from war zones [1]. Research conducted in some marginalized ethnic groups suggests that being a member of a minority ethnic group can be an independent risk factor for TB. That is, being poor increases the risk of TB, but being poor and a member of an ethnic minority increases the risk even further [2].

In general, Roma people are different from poor people of the major ethnic groups because they are more likely to remain poor, often live in ghetto-like settlements of cramped housing far from health services, and routinely experience discrimination from the healthcare system and other state services [3].

Almost all academic and gray literature agrees on three points: (1) there is little data about the health status of the Roma population; (2) the little data available indicate gross disparities between the Roma population and the major ethnic groups; (3) poor health among the Roma population is closely associated with the fact that they comprise a disproportionate percentage of the poor population [2].

Information about the health status of the Roma population is often not based on solid assessments. Some studies have reported higher rates of type two diabetes, coronary artery disease and obesity among Roma adults [4].

In Serbia, limited data show that the health status of the Roma population is far worse than that of the general population. The causes of this situation are closely related to poverty and exclusion: the unemployment rate among the Roma population is four times higher than the average in Serbia, and 32.5% of the Roma population have none or less than 4 years of education [5]. Furthermore, poor living conditions and discrimination in accessing health care services have an additional negative impact on the *health *of the Roma population. Consequently, TB could be a greater problem in the Roma population than in the general population in Serbia. Recent studies have highlighted the importance of increasing awareness about TB and reducing the associated stigmas to reduce the incidence of TB and enable earlier diagnosis and effective treatment [6,7].

This study was performed to investigate the knowledge and beliefs about transmission, symptoms and treatment for TB, as well as attitudes towards patients with TB among the Roma population in Belgrade.

## Methods

A total of 24 Roma people aged 19-55 years participated in three focus-group discussions. We planned three focus groups with 6-8 participants in each group for ease of facilitation, to give each participant an opportunity to contribute. We considered what might be the most successful way to achieve good recruitment balanced with cost and time available. Groups were selected according to living conditions in their communities. The first group included participants from the community with worst living conditions (slums). The second group included participants from the community with living conditions similar to the general population in Belgrade. The third group included participants with living conditions of a standard between groups 1 and 2. Researchers approached people that satisfied predetermined criteria (living in selected Roma community, aged 19-55) and provided them with information about the aims of the research and asked them if they were interested to contact the researchers at certain time and place in the community. At the initial contact with the researcher further information was given about likely dates and venues, transport arrangements, and that refreshments were to be provided. All groups comprised three males and five females. Considering that the participants are selected according to the previously chosen criteria, the opinions expressed in the discussion should be considered typical for the particular segment of the population only.

The focus-group method was considered to be appropriate for exploring the knowledge and beliefs about TB transmission, symptoms and treatment, opinions on appropriate preventive measures, and attitudes towards people with TB among the Roma population. The method does not seek to quantify data, but does try to explain certain phenomena to establish relevant hypotheses for further investigation [8].

The three focus groups addressed five main topics: knowledge of modes of transmission; knowledge and beliefs about symptoms of TB; knowledge and beliefs about effective treatment for TB and opinions about the most appropriate measures for TB prevention; main sources of information about TB; and attitudes towards patients with TB. Questionnaire for conducting focus groups discussion was prepared (Additional file 1)

Each focus-group session lasted 90-120 minutes. A trained psychologist with experience in moderating focus groups chaired the sessions in a non-directive manner. An observer participated in the sessions and her task was to record discussions as well as to ask complementary questions if any topics were not covered. Immediately after each session, the moderator, observer and researchers (authors) discussed the findings.

The sessions were recorded and transcribed verbatim. Data were analyzed and categorized into the objective or topics determined in advance. First, the interviews were read to become familiar with the text. The next step was to identify key issues in the text related to the topics. In the third step, the key issues were sorted according to the five central topics. Finally, the session data were arranged into new categories within each topic. All three authors discussed the categorization to achieve consensus about the content and the categories.

All participants in focus groups were informed about scope and objectives for the study and explicitly agreed to participate in the study.

The study was part of the Ministry of Health of the Republic of Serbia project "Control of tuberculosis in Serbia", supported by the United Nations Global Fund to Fight HIV/AIDS, TB and Malaria.

Ethical approval for the study was obtained from the Ethical committee of the School of Medicine, University of Belgrade.

## Results

All participants knew that TB is a pulmonary disease that can be contagious. The participants cited laboratory blood analysis as the most appropriate method to establish if someone is contagious.

"There are contagious and non-contagious forms of TB. Some blood analysis could be performed and it tells you all. Blood analysis shows whether it is contagious or not. If it is contagious, everyone living with you must give the blood for analysis, so it can be seen if they are infected."

Participants (especially those having someone close with TB) had heard of the term cavity and knew that patients with cavity are contagious.

### Knowledge of modes of transmission

Saliva and mouth-to-mouth contact were the most frequently mentioned modes of transmission.

"TB could be obtained if you drink from the glass or if you eat from the plate used by a TB patient. If we drink or eat after the patient some saliva stays on the spoon, the saliva contains TB bacteria and is transferred to our body so we get infected."

Participants thought that TB could be transmitted in the blood and among family members with the same blood type. Some of them even thought that TB could be hereditary. For example:

"If my mother has TB, I am more likely to get the disease than my wife. My mother and I have the same blood type."

"There was a man with TB. After a while his sister got TB as well. Maybe they got it from each other because they have the same blood type. As I heard it could be obtained through blood, it is hereditary."

Opinions about whether TB could be transmitted through hand shaking varied, some participants thought that it was a possible mode of transmission but others did not. Those who thought that it is not transmitted by hand shaking mentioned that they had shaked hands with a person with TB and still avoided getting TB themselves.

"I am not informed that TB could be transmitted through handshake. Even AIDS could not be transmitted that way. If I didn't get it from my sister it could not be transmitted that way."

Those who thought that shaking hands was a possible mode of transmission explained their views in the following way.

"It is possible to get TB through hand shakes. If I cough out bacteria in my hand, because I put my hand on the mouth when I cough, TB bacteria stay on my hand, and if I hand shake with you, bacteria pass on your hand, you touch your mouth and that's how it is transmitted."

During the session some participants mentioned rats as possible vectors for TB transmission.

"There are a lot of rats, big as cats, they bite. It is possible that rats transmit TB, they put there noses everywhere, in garbage and then pass over our dishes because we do not have closets in which to hide them."

### Factors contributing to TB occurrence

Participants mentioned poor living conditions, low quality and lack of food, and stress as factors contributing to TB.

"We are 12 in the house and I have only 20,000 dinars per month, about 250 euros. It is not nearly enough for 12 persons so we must eat something that I take from the garbage container. It is the source of all infections."

"TB is acquired from bad food, nervousness and worries. Yes it is; when you worry too much then disease appears."

The participants thought that, if untreated, the common cold and pneumonia could lead to TB. Furthermore, the participants thought that, if untreated, TB could develop into lung cancer.

### Knowledge about symptoms

Participants quoted: chest pain, cough, haemoptysis, loss of appetite, loss of weight, weakness and sweating as symptoms of TB.

"First symptoms are chest pain, not ordinary pain, more like stubbing. Also, you feel loss of appetite, you need more sleep, then you feel loss of strength and loss of weight. If one recognizes in time it is possible to recover."

"When you cough black content is expectorated from the lungs, like coal; also blood is constantly expelled through coughing."

Participants highlighted that it is hard to distinguish TB from other pulmonary diseases such as viral pneumonia and asthma. They emphasized haemopthysis as indicative of TB.

"It is hard for me to say if someone has TB. If you have chest pain, it could be that you have some virus, flu, asthma or pneumonia, it is not always TB. But you must take care. It is TB if you see someone coughing out blood or has bad pain in the lungs."

### Knowledge about treatment

Roma individuals mentioned that, in general, Roma people do not visit a doctor until the symptoms of the disease are so severe that they are unable to work

"*We Roma do not go to see a doctor while we can work; only when we lay in bed do we ask for doctor's help*". Regarding the effectiveness of treatment, some participants thought that TB is a very serious, lethal disease, whereas others thought that it is an unpleasant and long-lasting disease but that it is now curable owing to medical advances.

"TB is dangerous but curable. You take antibiotics, doctor tells you what to do, and than you can be cured. It is easier nowadays than it was before."

Some believe that although it is possible to treat TB, it is not totally curable. They explained that even after treatment is completed, some kind of scare stay on lungs and the lungs are vulnerable. The treatment is long lasting, and in some cases can last for 10 years.

"TB is a really dangerous disease. It is lethal if not treated. Even when treated, it lasts for a long time. My cousin stayed in hospital for 15 months. He was young, only eighteen. He probably didn't go to doctor in time. He barely survived."

Participants believe that effective treatment should include: resting, taking prescribed treatment, inhaling fresh air and eating "strong" food such as bacon and pork. Participants have very strong beliefs that "strong" food such as bacon, pork, sausages, fats and cream cheese is very good for preventing TB and could be as important as antibiotic treatment.

"Care for TB patient consists of resting, it is necessary take drugs prescribed by doctor. We gave her eggs, honey, bacon and sausages; pork is obligatory. She was sent to the mountains by doctors, where the air is sharp and clean, which is needed for TB treatment."

They also use some folk medicines to treat TB.

"*You take lemon, chop it, put in honey and whole egg with egg shell, the acid dilutes the eggshell, you should eat one little spoon every morning. It is the best medicine*."

Some participants thought that patients must pay for prescribed medicines themselves, but others were aware that medicines prescribed for TB are free of charge. Those who had a close relative with TB were aware that medicines are free of charge.

"*I think that medicines are free of charge. My daughter did not have to pay for them*."

### Opinions about most appropriate measures for TB prevention

Improvements in hygiene, living conditions, including electricity, sanitation and water are considered as key factors for TB prevention among participants. *"I heard that the easiest way to get the disease is through saliva, so at home I have my own glass so does my wife and our children as well. It would be inappropriate to drink from the same glass."*

Participants emphasized that they find it hard to protect themselves from TB, as living conditions are poor. They emphasized the importance of appropriate diet and quality food. Such food, which is expensive, is almost unaffordable for the participants and is probably symbolic of a good quality of life.

"You should have strong food for lungs to function."

"*Where we live it is hard to protect yourself. We don't have sanitation or electricity, and the water supply contains chemicals from nearby."*

### Source of information about TB

The main sources of information are relatives and friends and, to a lesser extent, television.

"When I lived with my mother in law, she told me about everything because she has seen it all. Also, I have seen some of it myself. I have children; I had to be informed to know how to treat them."

"We do not have enough time to watch television or to read newspapers, we must work. We mostly know from the experiences of our neighbors."

Participants can recall campaigns about tobacco control and AIDS but do not recall seeing anything about TB in the general media. The participants would appreciate direct interaction with a doctor who could come and talk to them about preventing TB and, if they are infected, provide advice on how they could protect others from TB. Participants expressed a high level of trust in doctors and believe most of what doctors tell them personally. They think that doctors on television could advertise different things. Therefore doctors on television are trusted less than those seen personally.

"I would mostly believe my own doctor, he is treating me. How can I know what they are talking about on TV, it could be only advertising."

"Don't speak about newspapers, some people can't read."

"We know about TB. But it is from experience. I would like a doctor to come and tell us."

### Attitudes towards patients with TB

Participants would visit a close relative with TB even if they knew the patient was contagious. Participants emphasize that the Roma people are very close and they often help each other, maybe more than non-Roma populations.

"I would not be afraid to visit my father or sister if they were ill. It is normal to visit them, I wouldn't be afraid for myself, but I would worry for my children."

"We are all Gypsies, we protect each other. We wouldn't leave without helping any of us. We visit one when he is sick. It is a shame if another goes to visit and I don't. You are embarrassed. If someone were sick, even with TB, I would come with bottle of juice. We are always one for another, (for each other) solider."

## Discussion

Various health problems, such as severe acute respiratory syndrome, avian flu and HIV/AIDS (but not its association with TB), as well as other chronic problems, are reported in the media to a much greater extent than TB [9].

Participants identify cough as an important symptom of TB; however, they do not associate cough with transmission of TB. The most common belief among participants was that TB is transmitted via saliva containing TB bacilli or via blood. However, transmission via blood is considered to be due to being of the same bloodline, so TB is seen as a 'hereditary' disease. Such beliefs could lead to stigmatization of TB patients. Some studies have shown that women could be considered unsuitable for marriage if they or a family member has suffered from TB [10].

Some participants thought that shaking hands with an infected individual could transmit TB. A recent study in the general population in Serbia found some misconceptions about the possibility of transmitting TB through physical contact with a person with TB (8.3% thought it was possible) and through food and drink (4.0% thought it was possible). However, TB was not mentioned as being hereditary [11]. Some similar misconceptions such as blood transfusion, drinking water and food as means of trasmission and TB being an inherited disease were obtained in the study conducted in Croatia [12]. Interestingly, a study on the Aboriginal population in Canada found that some respondents belived that TB is hereditary, but no explainations for this belief were reported [13]. It has been argued that knowledge about the symptoms of TB and their early recognition could help in timely treatment and prevention of disease transmission [13,9]. Generally, the Roma individuals in the focus groups knew the most common symptoms of TB. However, some respondents confused TB with other pulmonary diseases such as asthma and pneumonia, which is in agreement with the findings of San Sebastian and Bothamley [14].

Regarding factors contributing to the occurrence of TB none of the participants mentioned smoking. It has been noted that smoking is often not recognized as significant risk factor for TB, as showed in study in Croatia [12]. It is of great importance as smoking is much more prevalent in Roma communities than in general population. Smoking should be specially addressed in all health education campaigns regarding TB in Roma communities.

Our participants were aware about the need for prescribed treatment and that TB could be a dangerous, even lethal, disease. In addition to prescribed medicines, participants mentioned some folk medicines. Most participants emphasized the importance of appropriate diet and inhaling fresh air as appropriate preventative measures.

Our analysis showed that relatives and friends were the most common information sources, which is in agreement with results obtained from focus groups with poor Roma individuals conducted in Bulgaria and Romania; these studies concluded that Roma people felt more comfortable sharing personal health information and questions with their own community than outside their community [2]. Most Roma individuals would prefer to receive information by talking directly to their doctor. By contrast, the Aboriginal population in the Canadian study mentioned pamphlets, workshops and television as preferred information sources to learn about TB [12]. A lack of confidence in healthcare workers, of which only a few are from the Roma population, has been reported in the literature [2]; however, our results indicate the opposite.

## Conclusion

We concluded that participants were aware of the seriousness of TB as well as some modes of transmission; although there were some misconceptions. An important finding was the confidence in doctors expressed by the Roma people. This indicates that doctors should not only see patients with active TB, but should also be more engaged in providing information about TB prevention.

## Key Messages

• As the Roma people have a tendency to delay contact with the health service, it is important that health services provide outreach activities in Roma settlements.

• It is necessary to improve knowledge about TB treatment among the Roma population. Therefore, physicians and other health professionals should investigate knowledge and attitudes about TB and provide accurate information about TB and its modes of transmission. This is particularly important because they are considered the most trusthworthy individuals by the Roma community.

• Special attention should be paid to the potential use of folk medicines that could be harmful or cause further delay in contacting healthcare services.

## Competing interests

The authors declare that they have no competing interests.

## Authors' contributions

DV participated in the conception and design of the paper, data gathering, analysis and drafting the paper.

LjNO participated in conceptualiseng the paper, data analysis, drafting and revising it critically.

All authors have read and approved the final manuscript.

## Pre-publication history

The pre-publication history for this paper can be accessed here:

http://www.biomedcentral.com/1471-2334/11/284/prepub

## Supplementary Material

Additional file 1 **Questionnaire for conducting focus group discussion on TB**. List of questions that were prepared by the research team, to be addressed in focus groups Click here for file
