# Supplementary material for: Knowledge and awareness of tuberculosis among Roma population in Belgrade: a qualitative study
Source: BMC Infect Dis. 2011 Oct 24;11:284. doi: 10.1186/1471-2334-11-284 (PMC3210101; doi:10.1186/1471-2334-11-284)
Supplement: Additional file 1 — Questionnaire for conducting focus group discussion on TB. List of questions that were prepared by the research team, to be addressed in focus groups [file 1471-2334-11-284-S1.DOC]

APPENDIX 1

Guidelines for discussion in focus groups

INTRODUCTION

1. Introducing moderator and researchers
2. Informing participants about discussion in focus group
3. Informing participants about their role in the discussion
4. Informing participants about the conditions and reasons for recording the discussion and providing guaranties of anonymity
5. Introducing participants (name, age, occupation, number of household members)

KNOWLEDGE

1. Have you heard about TB? What do you know about it? How would you explain what is TB to someone who doesn’t know about it? Which organ/organs are affected in TB?
2. Do you know what are the symptoms pof TB, how does it manifest? Any other symptoms?
3. What would make you suspect that you or someone close to you has TB? Is there any symptom or sign that would make you believe that it is certainly TB? Why?
4. Is TB contagious? How is it transmitted? How can you acquire TB? Why?
5. Do you think that you can affect whether you get TB or not? Why do think that?
6. What do you think you should do to reduce your risk of getting TB? Why? How can you prevent TB?
7. Do you think that you can get ill of tuberculosis if:
8. you live in the house with a TB patient? Why? How?
9. you shake hands with a TB patient? Why?
10. you work in the same room with a TB patient? Why? How?
11. you are in the bus or cinema with a TB patient, why? How?
12. if you are close with member of family of TB patient, why? How?

8. How would you say, is TB a dangerous disease? Severe, medium, not very dangerous? Why do you think?

9. What do you think, can TB be successfully treated? Why? If you think that it can be treated, do you know how? Must patient be in a hospital or it can be treated at home?

10. Is it possible to die of TB if it is not treated? Why?

11. Do you think that there are many people with TB in our country? Why do you think that?

12. What do you think add to the occurrence of TB? What are the circumstances that increase burden of TB? (If not spontaneously mentioned ask: poverty, bad hygiene, lack of medicines, ignorance, lack of information, weak health care…)

13. Do you think that those who are employed in public services should be tested for TB? Why is it important?

ATTITUDE TOWARDS TB PATIENT

1. Has anyone close to you had TB? Who?
2. (If yes) how did this person get TB? Have you paid him/her a visit? *If yes) have you been afraid that you could get YB yourself? Have you done anything to prevent it? What exactly have you done? Have treated him/her the same way as any other patient? What was different? At what have you paid attention? Why?
3. (If they have not pay a visit) why haven’t you paid him/her a visit? What were you afraid of? Do you think that you might get infected during that visit? Why? What do you think, how would your friends and neighbors react if found out that you had visited a TB patient? Do you think that they would change their attitude towards you? How? Why?
4. (If they have never had anyone close with TB) Would you visit someone close to you if he/her had TB? Why? Would you be afraid to get infected? What do you think, would you get infected if you paid him/her a visit? What measures would you undertake to avoid infection? What do you think, should a patient with TB go to work? Why?
5. Do you know what comprise care of a TB patient? How long does treatment last? Does TB have permanent consequences on ones’ health? Which are the consequences?
6. According to you, what comprises treatment for TB?
7. Do you know if medicines for treating TB are free of charge? Should they be free of charge? Why do you think that?

INFORMATION

1. How have you learned about TB? What are your sources of information about health? (if not spontaneously mentioned, ask: friends, school, work, newspapers, radio, TV,…)
2. Have you ever noticed a campaign/advertise regarding health or some health problems? What was it about? What did you remember from that campaign? Why? What did you like? Why? What you did not like?
3. Would you say for yourself that you know enough about TB? What else would you like to learn, what kind of information you lack?
4. How would you like to learn? What would be the source of information that you would mostly trust? What that one?
